# Genetics for all: Tri-directional research engagement as an equitable framework for international partnerships

**DOI:** 10.1016/j.xhgg.2022.100140

**Published:** 2022-09-12

**Authors:** Thalia Billawala, Toluwani Taiwo, Neil A. Hanchard

**Affiliations:** 1 Childhood Complex Disease Genomics Section, Center for Precision Health Research, National Human Genome Research Institute, National Institutes of Health, Bethesda, MD 20892, USA

## Abstract

Over the past 5 years, human genetics and genomics research has placed a greater emphasis on increasing diversity among research participants and study researchers as a means of expanding the reach of human genetics and the knowledge accrued by it. Within this context, international collaborations between investigators in well-resourced research-funded countries (RFCs) and those in research-underfunded countries (RUCs) have flourished, with the goal of recruiting more geographically diverse participant pools. Past harms to communities engaged in genetics research have underscored the importance of bi-directional relational engagements, in which researchers and communities work together to ensure ethical research practices and participant involvement*.* Successful collaborations in the global genomics space, however, are often dependent upon RUC stakeholder investigators and physicians, whose needs are frequently either excluded from existing models of bi-directional community engagement or conflated with that of the study community. Here, we advocate for building more equitable international partnerships through the empowerment of RUC stakeholder investigators—a tri-directional engagement model—that includes supporting, building, and validating the efforts of RUC investigators through training, access, and authorship. We highlight existing initiatives that serve as exemplars in this effort and offer a framework for the broader genetics community to support equitable models of international research partnerships while being mindful of practical challenges. The core concepts embodied augment ongoing efforts to diversify the field of human genetics and complement the long-term goal of genetics for all.

## Teaser

In this commentary, we propose a framework of 3rd party investigator empowerment to build research capacity and foster equitable international—a tri-directional engagement model. We acknowledge challenges in establishing this model while arguing that the benefits are essential to the vision of diversity, equity, and excellence in genetics research.

## Main text

**Figure 1:**
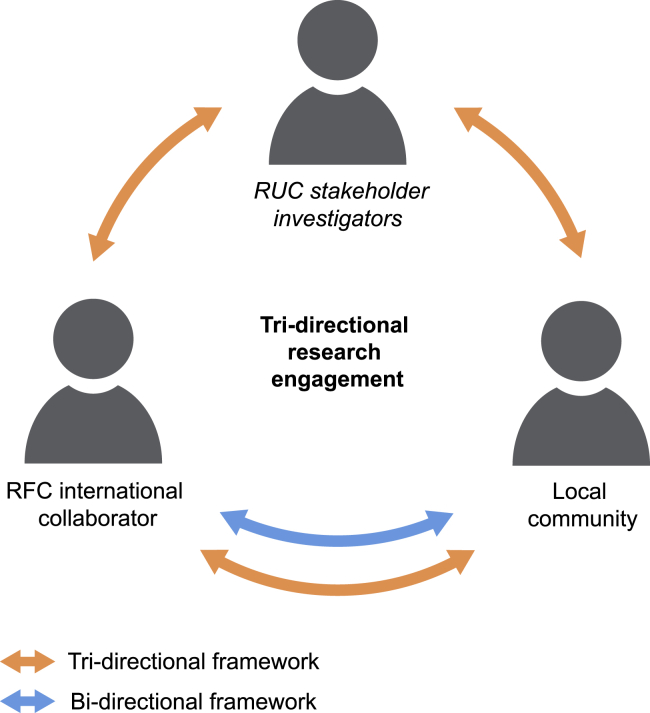
Tri-directional research engagement The tri-directional model entails an expansion of the current bi-directional collaboration between communities in research-underfunded countries (RUCs) and international researchers from research-funded countries (RFCs). It emphasizes the unique role an RUC stakeholder investigator or 3rd-party liaison plays in enabling engaged and equitable research in RUCs.

Over the past two decades, the need for international partnerships has become increasingly evident to address existing health inequities in both communicable and non-communicable disorders, and the human genetics community is no exception.^1^, ^2^, ^3^ With the growing acknowledgment of the dearth of ancestral diversity in human genetics and genomics studies, engaging diverse global cohorts through international partnerships has been touted as one means of expanding the “ancestral portfolio” of genetic studies.^4^ In practice, partnerships between genetic researchers and study communities that support such efforts are leveraged and sustained through a 3rd party community liaison.^5^ This role is typically filled by leaders from the study community, who are generally thought to gain similar benefits to the engaged community, including clinical and genetic knowledge, medical evaluations, laboratory results, and community empowerment, among others.^6^ In research-underfunded countries (RUCs), which include wealthy countries (based on gross domestic product) that lack sufficient monetary allocations toward research, the role of community liaison is often undertaken by RUC investigators and physicians alongside community leaders. Unfortunately, in international collaborations, these RUC partners often have inequitable representation and opportunities, especially when contrasted with their counterparts in research-funded countries (RFCs), where research efforts have a much more robust foundation of funding.^7^ Here, we proffer the need for tri-directional engagement that empowers stakeholder investigators in RUC countries alongside RUC communities and international collaborators in RFC countries as equitable partners in international genomics research to facilitate a more diverse genomics research portfolio and workforce (Figure 1).

### The emergence of bi-directional community research engagement

International research partnerships have historically been fraught with disparities that leave lasting effects on RUC communities. Within Africa, for example, international RFC researchers have been dubbed “vampires” in the past because of their method of “flying in” solely to take blood samples.^8^ Others have characterized RFC researchers working within Africa as performing “helicopter science” or taking “sample safaris” to obtain samples from local communities. In some situations, these perceptions of researchers have melded with cultural practices to create a systemic mistrust of medical research within the community.^9^^,^^10^ The collection of samples in these cases, and particularly their use without proper informed consent, created a precedent of exploitation of RUC communities. In the 1990s, investigators failed to disclose that blood samples from the Nuu-chah-nulth tribe, originally purposed for rheumatoid arthritis research, would be used in further studies.^11^ This action escalated concerns about research ethics in the scientific community, leading to a push for an ethical framework in genetics research.^12^ Subsequently, the genetics and genomics field, among others, expanded efforts in community engagement to emphasize more longitudinal and relational partnerships, even though perceptions of a paternalistic or neocolonial relationship remain in some cases.^6^^,^^13^ The foundation for this more equitable community engagement is bi-directional communication and discourse between RFC researchers and an RUC community, where ideas and goals are shared and contracted as part of a long-term relationship of mutual benefit.^14^

### Inequity between RUC and RFC investigators

In the international arena, the success of research partnerships is often dependent upon the efforts of “on the ground” investigators and clinicians, who are intimately involved in interfacing with ethics boards representing RUC communities, engaging and recruiting patient cohorts, and processing and accessing samples. These RUC stakeholder investigators often have academic career goals and aspirations that may differ from the motivations of an RUC community. As a result, international research partnerships impose a special consideration for RUC investigators that necessitates thinking beyond the classic bi-directional model toward a more inclusive tri-directional engagement.

The leadership structure in international research often favors investigators from RFCs over RUC investigators, as the former represent the primary source of funding; RUC investigators often have less of a say in the initiation, direction, and next steps of research.^15^ Further, most of the analyses that underwrite the academic output of the collaboration are spearheaded and carried out in RFCs.^16^ Consequently, RUC investigators face inequities from the outset of the study that often proliferate over the course of the partnership, leaving them without tangible opportunities for career advancement, even when promised otherwise.

The inequity between RFC and RUC investigators is evident in access to the resulting data. Modern genetic studies often result in the production of large and complex genomic data, including genome-wide association studies and high-throughput sequencing efforts. However, the downstream dissemination and utility of these data frequently either omits RUC investigators outright or creates data access limitations that systemically favor researchers from RFCs. For instance, when data generated in RUCs are transferred to RFCs for analysis or are “publicly” hosted in a manner that functionally restricts access by the same RUC investigators who generated the data (e.g., high-bandwidth requirements to download data, complex administrative bureaucracies to request data, or dumps of raw data without the tools or expertise to analyze it), RUC investigators are left with insufficient information needed to mount robust funding requests to continue their work or initiate new projects.^12^^,^^17^

A similar inequity is seen with potential commercial and 3rd-party uses of data (commercialization, grant procurement, etc.), which are not disclosed to RUC investigators or community members beforehand.^18^, ^19^, ^20^ This creates the potential for monetary gain for RFCs that excludes investigators, communities, and/or institutions in RUCs. Such circumstances could result in RUC groups incurring substantial charges to use information, software, or hardware they helped generate. Although known incidents of unethical data commercialization are not yet widespread, the potential for commercial exploitation looms large due to sizeable resource inequities and prevailing cultural factors (e.g., language barriers, lack of written documentation in lieu of expectations of sharing, or compulsory deference to RFCs).^7^ At the same time, genetic data protections have gradually moved toward more patient-driven and privacy-aware consent for generated data, which, paradoxically, also hinders research progress in RUCs by inhibiting RUC investigator access to the genetic and health information that they helped to collect.^21^^,^^22^

At the more egregious end, RUC investigators have also been omitted from authorship on publications, as their efforts to broker and manage complex sample collections may not meet the current academic bar for authorship.^8^^,^^23^ These omissions lead to a lack of subsequent opportunities for career growth and compound entrenched biases about the abilities and competence of RUC investigators.^24^ Furthermore, publication authorship is always fraught with the potential for controversy; the starting inequities of international research potentially amplify this. In RFCs, publications are the currency of academic and career advancement, and first and senior authorship positions are highly coveted.^25^ This incentivizes many overseas scientists in competitive research situations to pursue leadership on projects in RUCs, preventing RUC investigators from gaining these opportunities. Hedt-Gauthier et al. found significant disparities in representation and first authorship on papers that involved collaborations between African and RFC researchers. Out of approximately 7,100 total papers between 2014 and 2016, researchers from African countries made up 54% of all authors and 53% of first authors; however, if researchers from RFCs such as the USA, Canada, or Europe were collaborators, representation dropped by 10%–30% across total authorship and first authors (41% and 23%, respectively).^16^ In addition, systemic or unconscious biases can further exacerbate this inequity: a report on a 2014 lawsuit highlighted evidence of the prioritization of European researchers for promotions and training over Kenyan doctors in their collaborative enterprise.^9^^,^^26^ These examples underscore the dearth of research opportunities afforded to RUC investigators, especially as funding for research projects within RUCs is often project-based—once the project is completed, if the data are transferred and there are no remaining human or physical resources, there is little left to leverage.^15^ This approach perpetuates scientific disparities, further increasing the dependence of RUC investigators and their projects on RFC financial and academic support, thereby restarting the vicious cycle.

### Tri-directional engagement in international genomic research partnerships

To conduct truly equitable genetics research in the global context, therefore, there is a need to foster mutually beneficial partnerships, not only between well-funded RFC collaborators’ RUC communities, but between both groups and RUC stakeholder investigators who are usually the driving forces of such collaborations—tri-directional research (Figure 1). In its most effective form, equitable collaboration involves investigator-to-investigator connections between RFC and RUC investigators. This engagement should occur at all stages of research: development, design, implementation, analysis, and publication. Our understanding of this model builds from precedents set by ongoing initiatives. The Human Heredity and Health in Africa (H3Africa) Consortium—a program created to alleviate health disparities in Africa through genomic research—has been highly progressive in its emphasis on community engagement and core goals of nurturing the development of research infrastructures in Africa.^27^

**Figure 2:**
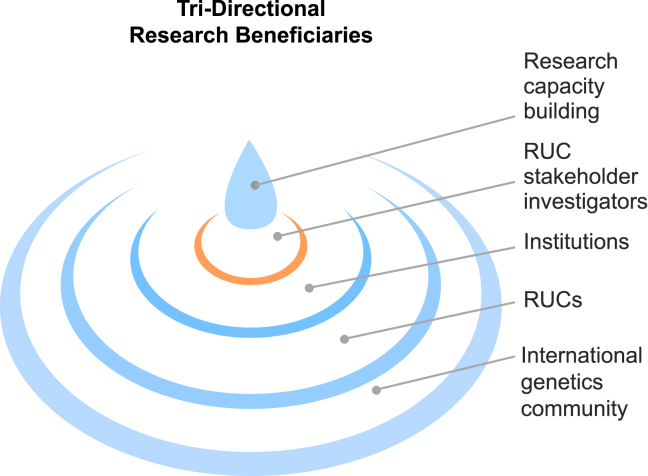
The potential ripple effect of supporting RUC stakeholder investigators By building research capacity in stakeholder investigators in RUCs, there is a ripple effect in benefits to RUC institutions, RUCs, and, ultimately, the international genetics community.

One of the aims of the Collaborative African Genomics Network (CAfGEN), an H3Africa program partnered with Baylor College of Medicine (BCM) in Houston, TX, USA, in which our lab participates, is to train African PhD students in genetics and genomics.^28^ This includes practical and theoretical educational experiences in high-throughput sequencing and bioinformatics.^29^ H3Africa’s and CAfGEN’s immediate goals are to increase research capacity and sustainability through a program of technology and expertise transfer, for which a key component is having RUC investigators serve as principal investigators (PIs) or co-PIs on collaborative projects. The ultimate goal is to equip African scientists with the skills and resources necessary for the initiation, implementation, and distribution of research projects, such that upon returning to their home countries, they can develop sustainable genomics research programs and investigate diseases affecting their communities. Although not formally documented, H3Africa graduates have already begun contributing to the genetics field as faculty at their respective universities and as facilitators of newly developed educational programs in genetics and genomics. Other benefits include developing human resources in genetics through long-term mentorship relationships (e.g., visiting researchers, peer-to-peer relationships), as well as didactic training and experiential learning opportunities in the form of seminars, H3Africa consortium meetings, and lectures in genetics and genomics (h3africa.org). The researchers who benefit from these collaborations then become the teachers and mentors in their home countries, further empowering their colleagues and institutions (Figure 2). Transfer of these skills may be directed to the RUC stakeholder investigators but more often benefits trainees and junior faculty within the department or center of the facilitating PI, thereby ensuring that a critical mass of well-trained and highly knowledgeable investigators comprise the next generation. Providing RUC investigators the opportunities and resources for training and skills development maximizes the potential for mutually beneficial collaborations and increases the number of thoroughly trained genomic scientists in RUCs—diversifying the genomics workforce.

The Malaria Genomic Epidemiology Network (MalariaGEN) consortium, a multi-continental initiative that seeks to eliminate malaria through genetics research, shares values of acknowledging community and RUC investigators’ interests, as well as protecting accumulated research data.^30^ Another research equity effort includes the Developing Excellence in Leadership, Training and Science (DELTAS) Africa program, which encourages the development and enhancement of research infrastructures while simultaneously offering mentorship and training projects across the African continent. The DELTAS program bolsters equitable partnerships by connecting tertiary and postgraduate students with “well-resourced universities, think tanks, and research institutions to strengthen capacity” (www.aasciences.africa/). Another notable example is the International Clinical Epidemiology Network (INCLEN) Trust, a research organization aiming to promote clinical epidemiology; through networking and capacity building activities, INCLEN Trust International empowers RUC investigators through international recognition and accreditation (www.hifa.org). This facilitates access to opportunities and collaborations beyond current projects, thereby establishing a sustainable and equitable model of conducting genetics research in RUCs. There are similarly placed efforts in other parts of the world; what ultimately underscores these programs is that they recognize RUC investigators not as conduits but as necessary and equal collaborators in international research engagements.

In addition to training opportunities, there are several other ways to empower RUC scientists within international collaborations. Increasing RUC access to “high impact” journals is one potential avenue, given that library services in RUCs are often unable to procure and maintain subscriptions to high-impact journals.^31^ Many initiatives, such as African Journals Online (AJOL), Wellcome Open Access, and Hindawi, seek to create open-access formats for investigators in RUCs, but RFC collaborators can further support these efforts by granting (even temporary) access to relevant scientific material.^32^, ^33^, ^34^ Allowing RUC investigators to participate in (and lead where appropriate) the development and conception of analyses, papers, and protocols can also promote skill enhancement and exposure. In addition, due to the influence of authorship on career progression, upfront and early discussions of authorship, including joint contributions, should be an important part of all collaborative research, and authorship for data provision and collection should be both discussed and encouraged. Early in the research engagement plans, similar thought needs to be put into approaches concerning disengagement from the research partnership—e.g., what happens to shipped samples at the end of the project period? How will future work be credited? International partnerships should also ensure that RUC investigators have functionally useful access to generated data—supported by training of data analysts and/or bioinformaticians in parallel with actual analyses. Such efforts are imperative to the sustainability of research in RUCs as they provide the human expertise and data needed to support future research grant proposals and publications. As the need for these types of international engagements grows, it is likely that the development of more streamlined engagement guidelines will follow. The suggestions above are designed to be illustrative of the kinds of activities that might characterize the path to equitable collaboration; however, RFC researchers should ultimately consider the articulated capacity-building needs of RUC stakeholders as paramount in fostering diverse collaborations.

### Challenges to tri-directional research collaboration

Arguably, the implementation of additional considerations in partnership developments between RUCs and RFCs might slow the progress of research due to more complex requirements for funding and/or extra time spent evolving partnerships with RUC communities and institutes. It may be that collaborations are already hindered by the difficulty of forming intercontinental (international) research connections, as there is no “standard operating procedure” for engagement in genetics research. Guidelines such as the “Montreal Statement on Research Integrity in Cross-Boundary Research Collaborations” contain useful recommendations, but they are by no means mandatory (wcrif.org). The lack of an enforceable framework for ethical collaboration, increased need for authorship in career advancement, absence of a common method for establishing collaborations, and the possible deceleration of research in the preliminary stages of this framework create significant challenges to tri-directional collaboration. Added to this, there is always the question of whether RUCs truly benefit from the adoption of technologies that may not fit with national priorities or cultural norms; this may be at the crux of potential collaborations that fail to materialize.

The counterargument is that this short-term view of deceleration is a major reason for the current “diversity gap” in genetics and genomics. As previously discussed, the existing model of collaborative research has not achieved the desired global good. Considerations of whether to engage RFC research technologies/paradigms should avoid being overly paternalistic in telling RUC institutions and stakeholder investigators what they may or may not need. A highly involved and active dialogue with RUC investigators and institutions to make costs and commitments clear while respecting RUC cultures, needs, priorities, and expertise is likely to be more fruitful in establishing the relationship. Although initial periods of protracted research might occur in the short term, by initiating good collaborative governance and equity, the likelihood of stronger long-term relationships—with the ability to respond rapidly and globally to changing research priorities—increases substantively.

### The role of the human genetics community

The genetics community as a whole can actively support more equitable research practices and tri-directional engagement in peer reviews, papers, and grant proposals. Multiple parties, including journals and grant funders, can collect metrics on RUC/RFC collaborative studies to monitor progress and develop plans to foster equity in these interactions. In peer reviews, for example, both reviewers and editors can ask for authors to clarify when work includes RUC communities but does not include RUC authors and insist on ethics statements that are explicit about RUC community involvement.^35^ In addition to expansion of current authorship guidelines, academic journals can actively support a broader definition of authors for studies that take place in RUCs.^23^^,^^35^ Where grant proposals involve international engagement, grant reviewers and funders could mandate that a portion of the project proposal specifically acknowledges RUC 3rd-party partners and delineates plans to ensure equity within their partnership. This would be similar to Sex and Gender Equity in Research (SAGER) guidelines, through which gender equity for research study participants is sought.^36^ Ethics review boards and committees within RUCs could be asking for evidence of an equitable research partnership between RFC collaborators and RUC investigators, using the aforementioned strategies as guidelines. Ultimately, the goal is to ensure that burgeoning collaborations intentionally identify mutual benefits and remain mindful of ethical ways to approach collaborations over the long term.

A similar remit exists around data; efforts have persisted to make genomic data publicly available without emphasizing that “available” is not synonymous with “accessible” or “usable,” particularly in RUCs.^19^ RFC investigators should be explicitly transparent about data rights in establishing collaborations with RUC investigators. At one end of the spectrum, this might involve binding agreements and direct statements about data usage in the initiating project, further projects, and any potential commercialization in collaboration agreements. At the most basic level, international genomics research should at least acknowledge where the potential for commercial (mis)use of genetic data exists in project protocols and documentation and include efforts to mitigate against this where it does. In concert, commercial transgressions or misuse of data should have consequences at the level of grant funding and journal publication. At the highest level, leaders within the genetic community can work to update existing data sharing policies and related laws to facilitate flexibility in data availability while still allowing for privacy. "Although fully private (i.e., unshared) genomic and health data can hinder progress by limiting the kinds of unique biological insights that can come from well-powered large-scale data analyses,^21^, ^37^ broadly open data that is suited to the analytical infrastructure and resources only available in RFCs, essentially excludes development of RUC investigators. Mindful considerations of this dichotomy, which could include longer publication embargos or collaborative use of public data, can still allow for progress on both fronts.

Historically, RUC investigators have acted as intermediaries in international collaborations; now, with global populations and diverse cohorts coming to the fore of genetics and genomics research, there is an opportunity to equitably empower RUC stakeholder investigators with knowledge transfer, skills development, and resources to propagate research engagement in RUCs. A tri-directional model of community engagement will be imperative to conducting the high-quality and ethically grounded global research needed to galvanize human genetics discoveries in the future.

## Web resources

AA Sciences, https://www.aasciences.africa/aesa/programmes/developing-excellence-leadership-training-and-science-africa-deltas-africa
H3Africa, https://h3africa.org/wp-content/uploads/2018/05/CE%20Revised%20Guidelines_Final_September%202017%20(1).pdf
HIFA, https://www.hifa.org/support/supporting-organisations/inclen-trust-international-clinical-epidemiology-network
WCRIF, https://wcrif.org/montreal-statement/file
